# Monkeypox in Dentistry: A New Opportunity for Research and Collaboration

**DOI:** 10.1016/j.identj.2022.11.007

**Published:** 2022-12-05

**Authors:** Frank Mayta-Tovalino, John Barja-Ore, Daniel Alvitez-Temoche

**Affiliations:** a CHANGE Research Working Group, Universidad Científica de Sur, Lima, Peru; b Research Direction, Universidad Continental, Lima, Peru; c Postgraduate Department, Faculty of Dentistry, Universidad Nacional Federico Villarreal, Lima, Peru

Dear Editor:

Monkeypox virus (MPX) is a new outbreak of an old disease now manifesting in many regions of the world.^1^ Caused by a zoonotic virus, it may be transmitted through the airborne route, although direct contact with affected lesions or contagious materials are the usual modes of transmission.^2^ In the recent outbreak, many of the cases have been identified as being in men who have sex with men, with lesions mainly in the genital regions.^3^ During 2022, as of October 19, the World Health Organization has reported in all regions a total of 73,437 cases of monkeypox and 29 deaths.^4^ Infected patients usually present with headache, chills, fever, sore throat, fatigue, muscle discomfort, lymphadenopathy, and oral and skin lesions that evolve into ulcerative pustules.^5^, ^6^, ^7^ Early warning signs of the disease often appear as spots and ulcers on the oral mucosa before the typical skin lesions.^6^

Prevention and control of MPX transmission in dental practice necessitates maintaining the extant robust standard infection control measures applied with all patients.^6^ Current recommendations are to avoid dental treatment of patients with MPX who can still transmit the virus and, if essential, to provide such care in an isolated environment with the appropriate protective measures for the dentist and their team.^7^

We recently conducted a systematised Scopus search on the global scientific publications on MPX in dentistry. It was evident that a few publications on the latter topic were available in this emerging field. These were, notably, in 2 major scientific journals of high impact: the *International Dental Journal* (a single publication) and *Oral Diseases* (2 publications). Samaranayake, Lakshman Perera (h-index 97; Google Scholar) and Lo, Muzio Lorenzo (h-index 56) were the major contributing authors of these, and additionally, most of the authors were from Brazil and India.

**Figure 1:**
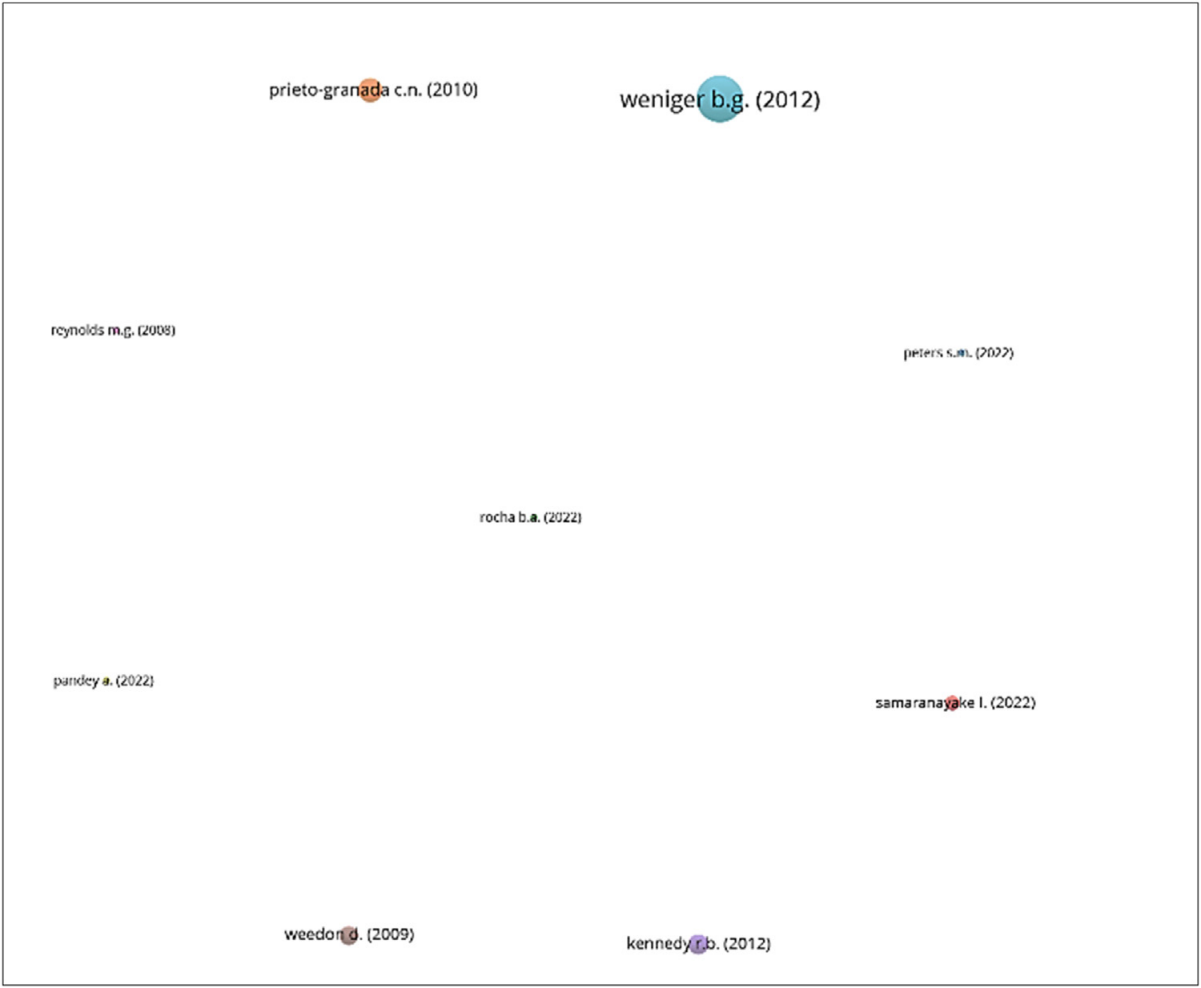
Citations by author on Monkeypox in dentistry.

As MPX is a relatively new subject beginning to be explored by the scientific community in dentistry, researchers have an opportunity now to contribute actively to the generation of new knowledge on MPX so that, over time, a better visualisation of the dynamics of publications and collaboration between authors and research institutions could be deciphered.

In conclusion, MPX appears to be a serious disease, and dentists should be aware of the premonitory signs of the disease in the oral cavity and how to treat patients with suspect lesions, as per the local guidelines. Oral manifestations of the disease in all who present with MPX should be recorded and published to generate a comprehensive account.

## Conflict of interest

None disclosed.
